# Use of the Carolina HPV Immunization Attitudes and Beliefs Scale (CHIAS) in Young Adult Women

**DOI:** 10.1371/journal.pone.0100193

**Published:** 2014-06-19

**Authors:** Amanda F. Dempsey, Andrea Fuhrel-Forbis, Sara Konrath

**Affiliations:** 1 Children’s Outcomes Research Program, University of Colorado Denver, Aurora, Colorado, United States of America; 2 Institute for Social Research, University of Michigan, Ann Arbor, Michigan, United States of America; University of Saskatchewan, Canada

## Abstract

**Background:**

Validated measures that can accurate describe young adults’ HPV vaccination attitudes and how these relate to vaccination intention and receipt are needed for developing interventions to improve low HPV vaccination levels. The Carolina HPV Immunization Attitudes Scale (CHIAS) is a validated measure of these outcomes that was originally designed for parents.

**Objective:**

To assess the performance of the CHIAS among young adult women using an exploratory factor analysis.

**Methods:**

A convenience sample of 139 young adult women (age 18–26 years) were given the CHIAS measure at baseline. Factor analysis was used to determine attitudinal factor groupings and the association of these factors with HPV vaccination intention. A 6-month follow up assessment examined the stability of the CHIAS over time and the association of baseline vaccine factors with vaccine receipt.

**Results:**

Five factors loaded on to the CHIAS in young adults - “Barriers,” “Harms,” “Effectiveness,” “Risk Denial” and “Uncertainty,” - which was similar to the factor loadings of CHIAS for parents. “Harms” was the factor most consistently associated with vaccination intention at all time points assessed. Only 5 women had received or made an appointment to receive the vaccine at the 6-month follow-up.

**Conclusions:**

In terms of categorizing HPV vaccination attitudes, the CHIAS appears to have similar performance among young adults as in parents. However, additional studies are needed to assess the utility of the CHIAS for predicting HPV vaccine receipt among the young adult population.

## Introduction

Vaccines against human papillomavirus (HPV) represent a remarkable opportunity for the primary prevention of cervical cancer and other HPV-related diseases. Despite these health benefits, HPV vaccination among young adults in the U.S. is significantly lower than national goals. [1] Compared to adolescents, [2] young adult women have substantially lower HPV vaccine utilization, with national estimates indicating that as of 2012, only 34.5% of women ages 19–28 years had received at least one dose in the 3-dose HPV vaccine series. [3].

Interventions to improve HPV vaccine utilization among young women have been hindered by limited understanding of the factors that influence vaccine acceptability, intention, and ultimately vaccine utilization among this population. [4] Though there have been several studies on young women’s attitudes about HPV vaccination, [5]–[7] a validated measure that can accurately categorize attitudes about the vaccine and predict vaccination intention and receipt is not yet available. However, such a measure has been developed for parents making decisions about HPV vaccination for their adolescents and is called the Carolina HPV Immunization Attitudes Scale (CHIAS). [8]–[10].

The CHIAS, developed by McRee et al, was originally evaluated among a regional sample of parents in North Carolina. [8] Analysis of this 16-item scale resulted in the identification of 4 factors (Harms, Effectiveness, Barriers, Uncertainty) that categorized parental attitudes about HPV vaccines. Subsequent longitudinal analyses demonstrated the stability of these factors to describe HPV vaccination attitudes over time. [9] Three the four factors (all but Effectiveness) that were associated with parent HPV vaccination intention also predicted actual HPV vaccine utilization by these parents’ adolescents. When the CHIAS was examined among a nationally-representative sample of parents, a very similar factor structure resulted, suggesting that the CHIAS is a robust measure of parental attitudes about the vaccine. [6] Unfortunately, this national study did not assess the association between the CHIAS factors and vaccine utilization.

Having a similarly robust, standardized measure of HPV vaccination attitudes for young women would be useful for developing and examining interventions to improve HPV vaccine uptake among this population. Therefore, the goal of this study was to examine the factor structure of the CHIAS when applied to a sample of young adult women. The specific objectives were: 1) to compare the factor structure that results from young women’s use of the CHIAS to that reported previously for parents, and 2) to evaluate the association between the CHIAS factors and young women’s HPV vaccination intention and utilization.

## Materials and Methods

### Study Design

We conducted a cross sectional survey of 139 college-aged women that was implemented from October 11, 2011 to November 1, 2012. This survey was part of a larger study aimed at evaluating the longitudinal impact of different educational materials on HPV vaccination intention and receipt (end result was no difference in these outcomes by educational group), and on hormonal stress responses to those materials (manuscript in preparation). The focus of this manuscript is on responses to CHIAS items specifically.

### Ethics Statement

This study was approved by the Institutional Review Board at the University of Michigan. Written informed consent was received from all participants.

### Participants

Participants were recruited via a Psychology participant pool and flyers posted on campus and in the local town advertising a study about HPV vaccines. Eligibility criteria for participation included being a female aged 18–26 years and not yet having received any doses in the HPV vaccine series. Upon arriving to the study lab and providing informed consent, participants received a paper version of the baseline survey to complete in a private cubicle in the lab. Follow-up surveys were emailed to participants and administered via Qualtrics with up to 2 prompts for non-respondents.

### CHIAS Items

We used all 16 items described in the original CHIAS [11] and also included one additional item from a modified version of the CHIAS that had been validated previously among a national sample of parents (“*HPV vaccination is not really necessary because Pap smears can be done to make sure cervical cancer doesn't develop”*). [6] For each item derived from previous versions of the CHIAS, wording was changed to reflect a young adult, rather than parent, perspective (i.e. “*Other parents in my community are getting their daughters vaccinated*” becomes *“My friends are getting the HPV vaccine*”). A side-by-side comparison of the original CHIAS items and the modified items used for this analysis is described in Table 1. All responses were assessed using an 11-point Likert scale (with anchors at 0, 5, 7 and 10 of “strongly disagree,” “somewhat disagree,” “somewhat agree” and “strongly agree”; or anchored at “extremely ineffective, “somewhat ineffective,” “somewhat effective” and “extremely effective”) and were coded such that higher values corresponded to stronger agreement with the statement and less agreement with or endorsement of HPV vaccination. Five items were reverse-coded.

**Table 1:** Comparison of Original CHIAS items and Modifications Used for this Study.

Original CHIAS	Modified CHIAS used in this Study
The HPV vaccine might cause short term problems, like fever or discomfort.	I think the HPV vaccine might cause short term problems, like fever or discomfort.
The HPV vaccine is being pushed to make money for drug companies.	The HPV vaccine is being pushed to make money for drug companies and/or doctors.
The HPV vaccine might cause lasting health problems.	I think the HPV vaccine might cause health problems in the future.
If a teenage girl get this HPV vaccine, she may be more likely to have sex.	I think that getting the HPV vaccine makes it more likely for someone to have sex.
I think the HPV vaccine is safe.	I think the HPV vaccine is safe.
[Child’s name] is too young to get the HPV vaccine.	I think I am too young to get a vaccine for a sexually transmitted infection like HPV.
How hard do you think it would be to find a provider or clinic where youcan afford the vaccine?	It would be hard to find a provider or clinic where I could afford the HPV vaccine.
How hard do you think it would be to find a provider or clinic that iseasy to get to?	It would be hard to find a provider or clinic that would be easy to get to for getting vaccinated against HPV.
How hard do you thin k it would be find a provider or clinic that has thevaccine available?	It would be hard to find a provider or clinic that has the HPV vaccine available.
I am concerned that the HPV vaccine costs more than I can pay.	I am concerned that the HPV vaccine costs more than I can pay.
How hard do you think it would be to find a provider or clinic where youdon’t have to wait long to get an appointment?	It would be hard to find a provider or clinic where I don’t have to wait a long time to get an appointment to be vaccinated.
How effective do you think the HPV vaccine is in preventing genital warts?	How effective do you think the HPV vaccine is in preventing genital warts?
How effective do you think the HPV vaccine is in preventing cervical cancer?	How effective do you think the HPV vaccine is in preventing cervical cancer?
I don’t have enough information about the HPV vaccine to decide whetherto give it to [child’s name].	I have enough information about the HPV vaccine to decide whether to get it.
The HPV vaccine is so new that I want to wait a while before deciding ifmy daughter should get it.	The HPV vaccine is so new that I want to wait a while before deciding if I should get it.
Other parents in my community are getting their daughters theHPV vaccine.	My friends are getting the HPV vaccine.
–	HPV vaccination is not really necessary because Pap smears can be done to make sure cervical cancer doesn’t develop.

### Outcome Variables

HPV vaccination intention and receipt were assessed as outcome variables. HPV vaccination intention was measured with two items that asked participants about the likelihood of getting the vaccine “*today if it was available for you,*” or “*within the next 6 months*” using a previously-published 11-point vaccination intention scale. [12]–[14] This outcome was asked at baseline, and at a 6-month follow-up survey. Vaccination “receipt” was determined by self-report at the 6-month follow-up survey and was defined as a positive response to at least one of two questions: “*Since you were in the lab for the first part of the study 6 months ago, have you received any doses (shots/injections) of the HPV vaccine?*” *(yes/no),* and “*Have you made an appointment to get the vaccine?*” *(yes/no).*

### Statistical Analysis

An exploratory factor analysis of the baseline CHIAS items was conducted using principal components analysis with oblique rotation method (as factors were assumed to be correlated). Factors meeting the Kaiser criterion (Eigenvalues ≥1.0) were retained. Non-weighted factor scores (consistent with previous CHIAS assessments) [8], [9], [13] were created for each respondent by calculating the mean of the responses to all items loading onto each factor. Cronbach’s α coefficient was used to evaluate the internal reliability of each factor grouping. We performed our factor analysis forcing a four factor solution, in order to assess how well the original CHIAS factor groupings applied to this population, and also under an “unrestricted” factor strategy.

Linear and logistic multivariable regression models were used to examine the association between the different factor groupings with vaccination intention and uptake, respectively. Each model included the 5 factors derived from the exploratory factor analysis, but no other covariates were added given our relatively small sample size (n = 139). For all analyses, *p*-values ≤0.05 were considered statistically significant. All analyses were performed using SPSS 20).

## Results

### Study Sample

Of the 139 participants who completed the baseline survey, 98 (70.5%) also completed the 6-month follow-up survey. As shown in Table 2, at baseline 41% of respondents were in a current sexual relationship, and nearly all had heard of HPV and knew a vaccine was available. Only a small proportion of respondents indicated they had ever experienced an HPV-related disease (2–5%).

**Table 2:** Sample Characteristics of College-Aged Females in Study at Baseline.

Variable	Sample N = 139
Mean age, yrs (range)	20 (19–25)
% Currently in sexual relationship*	41%
Lifetime number of sexual partners (range)*	1 (0–8)
Relationship status (%)	
Single and not dating	55%
Dating more than one person	1%
In a relationship with one person only (dating, engaged, married)	44%
% Ever heard of HPV	94%
% Knew there was an HPV vaccine available	98%
% Ever diagnosed with genital warts	2%
% Ever diagnosed with an abnormal Pap smear	5%
% Never diagnosed with a sexually transmitted infection	99%

*Sexual partner and sexual relationship were defined as having any intimate genital contact.

### Factor Structure among Young Women

When using a forced 4-factor solution, the exploratory factor analysis had good consistency in factor groupings with the original CHIAS for the “Barriers” and “Harms” factors. However, the statements loading to the “Effectiveness” and “Uncertainty” factors were markedly different from the original CHIAS under this solution strategy. When the analysis was repeated removing the restriction to 4 factors, the exploratory factor analyses demonstrated 5 factor groupings (Table 2), which included the four original CHIAS factors plus an additional factor which we labeled “Risk Denial”. Three of the factors, “Barriers,” “Harms,” and “Effectiveness,” showed good internal reliability (Cronbach’s alpha 0.74–0.91, Table 3). The internal reliability of the other two factors, was considerably lower (0.43–0.49, Table 3).

**Table 3:** Factor Profiles of the CHIAS Assessed at Baseline and at 6-month Follow-up.

Factor Items	Barriers	Harms	Effectiveness	RiskDenial	Uncertainty	Eigenvalues
It would be hard to find a provider or clinic that would beeasy to get to for getting vaccinated against HPV.	**0.924**	−0.004	−0.034	0.077	0.150	4.111
It would be hard to find a provider or clinic where I couldafford the HPV vaccine.	**0.913**	0.050	−0.058	0.068	0.156	3.697
It would be hard to find a provider or clinic that has theHPV vaccine available.	**0.890**	−0.072	0.037	0.161	0.218	1.426
I am concerned the HPV vaccine costs more than I can pay.	**0.873**	0.020	−0.064	0.025	0.132	1.190
It would be hard find a provider or clinic where I don’thave to wait a long time to get an appt. to be vaccinated.	**0.801**	−0.067	−0.001	0.039	0.121	1.050
I think the HPV vaccine may cause health problemsin the future.	−0.072	**0.897**	0.251	0.298	0.061	0.893
I think the HPV vaccine is unsafe.	−0.017	**0.836**	0.423	0.267	0.195	0.838
I think the HPV vaccine might cause short term problemslike fever or discomfort.	0.067	**0.717**	0.180	−0.060	−0.074	0.733
The HPV vaccine is so new that I want to wait a whilebefore deciding if I should get it.	−0.141	**0.694**	0.068	0.500	0.039	0.649
I think the HPV vaccine is being pushed to make moneyfor drug companies and/or doctors.	0,033	**0.559**	0.289	0.339	0.150	0.533
How effective do you think the HPV vaccine is inpreventing genital warts? If you don’t know, makeyour best guess.*	−0.167	0.209	**0.869**	0.055	0.105	0.451
How effective do you think the HPV vaccine is inpreventing cervical cancer?*	0.033	0.402	**0.843**	0.265	0.033	0.431
I think that getting the HPV vaccine makes it more likelyfor someone to have sex.	0.022	0.121	0.122	**0.738**	0.129	0.345
HPV vaccination is not really necessary because Papsmears can be done to make sure cervical cancer doesn’t develop.	0.124	0.200	0.046	**0.659**	0.070	0.267
I think I am too young to get a vaccine for a sexuallytransmitted infection like HPV.	0.112	0.301	0.151	**0.578**	−0.164	0.172
I have enough information about the HPV vaccine todecide whether to get it.*	0.182	0.087	−0.011	0.115	**0.868**	0.149
My friends are getting the HPV vaccine.*	0.237	0.099	0.455	−0.045	**0.639**	0.065
Factor Mean (SD)	1.60 (1.85)	4.87 (2.04)	4.10 (1.36)	2.61 (1.78)	5.30 (2.32)	–
Factor Cronbach Alpha	0.92	0.81	0.74	0.49	0.43	–

*Items were reverse-coded to maintain consistency, with higher values corresponding to less support for HPV vaccines.

Bolded items demonstrate factor groupings.

### Association of Baseline Factors with Vaccination Intent Assessed at Baseline

As shown in Table 4, all the factors except Uncertainty were associated with vaccination intention when assessed at baseline for the outcome of willingness to receive the vaccine if it were available “today.” Higher perceived difficulty in accessing the vaccine (Barriers factor) was associated with *increased* vaccination intention whereas increased concern about harms (Harms factor), lower perceived effectiveness (Effectiveness factor) and stronger risk denial attitudes (Risk Denial factor) were associated with lower vaccination intention. Interestingly, when assessing vaccination intention for the coming 6 months, the Barriers and Risk Denial factors were no longer associated with this outcome (Table 4).

**Table 4:** Relationship Between Factors and Baseline Intentions for HPV Vaccine***.

	Baseline Vaccination Intention for “today”*		Baseline Vaccination Intention for the “next 6 months**	
Baseline Factors	Standardized Beta Coefficients	*p*-value	Standardized Beta Coefficients	*p*-value
**Access**	**0.33**	**0.002**	0.12	0.29
**Harms**	−**0.53**	**<0.001**	−**0.31**	**0.007**
**Effectiveness**	−**0.35**	**0.02**	−**0.38**	**0.02**
**Risk Denial**	−**0.27**	**0.02**	−0.24	0.06
**Uncertainty**	−0.06	0.46	−0.04	0.69

*Assessed at baseline by measuring response to the question “If the HPV vaccine was available for you today, how likely would you be to get vaccinated?”.

**Assessed at baseline by measuring response to the question “How likely are you to get the HPV vaccine within the next 6 months?”.

***Multivariable model that includes all factors listed.

Bolded values highlight statistically significant relationship.

### Association of Baseline Factors with Vaccination Intent Assessed at Follow-up

Comparing Tables 4 and 5, there were notable differences in the relationship between the factors and vaccination intention when participants were assessed at baseline versus at the 6-month follow-up. In contrast to the baseline assessment (Table 4), only Barriers and Harms were associated with vaccination intention for “today,” and only Harms was were associated with vaccination intention for the coming 6 months when assessed at the follow-up survey (Table 5).

**Table 5:** Relationship Between Baseline Factors and 6-month *Follow-up* Intentions for HPV Vaccine^¥^ *** ^.^

	Follow-up Vaccination Intention for “today”*		Follow-up Vaccination Intention for the “next 6 months**	
Baseline Factors	Standardized Beta Coefficients	*p*-value	Standardized Beta Coefficients	*p*-value
**Access**	**0.18**	**0.025**	0.11	0.18
**Harms**	**−0.42**	**<0.001**	**−0.35**	**<0.001**
**Effectiveness**	−0.05	0.57	−0.11	0.21
**Risk Denial**	−0.11	0.17	−0.12	0.15
**Uncertainty**	−0.07	0.39	−0.01	0.95

*Assessed at follow-up by measuring response to the question “If the HPV vaccine was available for you today, how likely would you be to get vaccinated?”.

**Assessed at follow-up by measuring response to the question “How likely are you to get the HPV vaccine within the next 6 months?”.

*** Multivariable model that includes all factors listed.

¥ Follow-up survey occurred 6 months after baseline. N = 98.

Bolded values highlight statistically significant relationship.

### Association of Baseline Factors with Vaccination Receipt at Follow-Up

Only 5 out of 98 women (5.1%) completing the 6-month follow-up assessment indicated that they had either received the HPV vaccine or made an appointment to get it since the baseline assessment. These low numbers precluded us from being able to perform any meaningful statistical analyses on how well the factors identified using CHIAS predicted vaccination uptake.

## Discussion

Measures that reliably predict HPV vaccination intention across populations and over time could help facilitate the development of effective interventions to improve HPV vaccine uptake. The original CHIAS [15] was tested among parents of adolescents and found to be a useful tool to categorize HPV vaccination attitudes, with each identified factor reliably predicting vaccination intention over time, and three of the four factors longitudinally predicting vaccination receipt. When we evaluated the factor loadings of the CHIAS among young adult women, we found the overall factor structure to be robust - there were significant similarities in the items loading to the Barriers, Harms, Effectiveness and Uncertainty factors between young women in our study and prior analyses of CHIAS in parents. However, in our study a new factor emerged from the CHIAS, which we termed Risk Denial. This new factor contained correlates of two statements that loaded to Harms in the original CHIAS (“I think that getting the HPV vaccine makes it more likely for someone to have sex” and “I think I am too young to get a vaccine for a sexually transmitted infection) in addition to the new item added for assessment in our study (“HPV vaccination is not necessary because Pap smears can be done to make sure cervical cancer doesn’t develop”). It was notable that 2 of the 3 items loading to the Risk Denial factor relate to low perceived risk of HPV infection or sequelae (vaccine non necessary because of Pap tests; too young to get a vaccine against an STI). Our results suggest that young women may have subtle differences in attitudes about HPV vaccines from parents of adolescents that could be important to consider for intervention to improve vaccine uptake among this population. Furthermore, our findings may indicate a heightened need to “convince” young women about their individual risk for HPV infection and disease. Moreover, the finding that items loading to the Harms construct appear to be consistent and reliable across populations, combined with the fact that in our study Harms is the most consistent predictor of vaccination intention both immediately and longer-term, suggests that interventions focusing on mitigating concerns about the vaccine’s harms may be a particularly effective educational strategy for increasing HPV vaccination among young adults.

An interesting finding from our study was the association between the Barriers factor and vaccination intention. When assessed at baseline, young adults with higher perceived barriers to accessing the vaccine had an *increased* vaccination intention if the vaccine were available “today.” In contrast, at baseline there was no association between Barriers and vaccination intention when intentions for the “next 6 months” were assessed as the outcome, or when vaccination intention was assessed for either time frame in the follow-up survey. This finding could signify that the young women in the study had a very literal interpretation of having “the vaccine available for you today.” Participants may have believed that they would have opportunity to get the vaccine in the study lab after taking the baseline assessment (which was not the case). If so, it is understandable that those with higher perceived barriers to accessing the vaccine would have a higher vaccination intention for a vaccine that might be immediately available, and that access would be unrelated to a vaccine dose theoretically available 6 months in the future, or when reassessed by email where “vaccinating today” by the study team was obviously not a realistic possibility. These findings suggest that coupling HPV vaccination education with immediate access to the vaccine may be an effective strategy to increase HPV utilization among young adults.

In the original CHIAS study in parents, [8] Harms, Effectiveness, Barriers and Uncertainty were all associated with vaccination intention, and all but Effectiveness was associated with actual vaccine receipt when vaccination status was assessed a year later. [9] In our study there were only 5 women who reported either getting the vaccine or making an appointment to get the vaccine between the baseline and follow-up assessments, making it difficult to draw conclusions about the interrelationship between CHIAS factors, vaccination intention and vaccine receipt in young adults – an unfortunate limitation of our study. Other limitations that are important to consider for this study are the relatively small sample size that was drawn from a limited geographic area, which impacts the generalizability of the results. In addition, participants involved in the study were exposed to one of four different educational materials immediately after their baseline assessment. While none of the interventions appeared to have impacted vaccination intention or receipt (manuscript in preparation) either when assessed immediately following the intervention or at the 6-month follow-up, it is possible that the variability of educational materials could have had subtle influences on the CHIAS factor loadings when assessed over time.

## Conclusions

CHIAS items appear to group into very similar factors when comparing parents making decisions about HPV vaccination for their adolescents to young women making the HPV vaccination decision for themselves, suggesting that the CHIAS is robust measure for categorizing HPV vaccination attitudes. However, the association of these factors with vaccination intention appears to differ between parents and young adults. Harms was the only factor that performed similarly between these two populations and also consistently predicted vaccination intention over a variety of time frames. This suggests that educational strategies focusing on mitigating perceived harms from the vaccine may have the widest influence and appeal across populations of different ages.
